# Stygofauna enhance prokaryotic transport in groundwater ecosystems

**DOI:** 10.1038/srep32738

**Published:** 2016-09-06

**Authors:** Renee J. Smith, James S. Paterson, Elise Launer, Shanan S. Tobe, Eliesa Morello, Remko Leijs, Shashikanth Marri, James G. Mitchell

**Affiliations:** 1 School of Biological Sciences, Flinders University, Adelaide, South Australia, 5001, Australia; 2 Department of Chemistry and Physics, Arcadia University, Glenside, Philadelphia, 19038, USA; 3 South Australian Museum, North Terrace, Adelaide, South Australia, 5001, Australia; 4 School of Medicine, Flinders University, Adelaide, South Australia, 5001, Australia

## Abstract

More than 97% of the world’s freshwater reserves are found in aquifers, making groundwater one of the most important resources on the planet. Prokaryotic communities in groundwater underpin the turnover of energy and matter while also maintaining groundwater purity. Thus, knowledge of microbial transport in the subsurface is crucial for maintaining groundwater health. Here, we describe for the first time the importance of stygofauna as vectors for prokaryotes. The “hitch-hiking” prokaryotes associated with stygofauna may be up to 5 orders of magnitude higher in abundance and transported up to 34× faster than bulk groundwater flow. We also demonstrate that prokaryotic diversity associated with stygofauna may be higher than that of the surrounding groundwater. Stygofauna are a newly recognized prokaryotic niche in groundwater ecosystems that have the potential to transport remediating, water purifying and pathogenic prokaryotes. Therefore, stygofauna may influence ecosystem dynamics and health at a microbial level, and at a larger scale could be a new source of prokaryotic diversity in groundwater ecosystems.

Prokaryotes in terrestrial subsurface environments, which include groundwater, account for 40% of the global prokaryotic biomass, with overall abundance estimates of 4–6 × 10^30^ cells^1^. These prokaryotic communities play a fundamental role in the turnover of biosphere energy and matter^2^,^3^, while also purifying groundwater^4^. Prokaryotic communities typically consist of a mixed consortium, which allows for rapid responses to environmental perturbations^5^,^6^,^7^. This rapid response to change means that microbial communities are often tracked as biological indicators^8^,^9^. Thus, many studies have focused on the advection transport of prokaryotic communities in groundwater to determine ecosystem health^9^,^10^.

The importance of prokaryotes in groundwater has highlighted the need for an improved understanding of the transport of microbial communities in subsurface environments^11^. Transport of microbes in the subsurface involves a host of complex physiochemical and biological parameters, including advection and prokaryotic motility^11^,^12^. Prokaryotes in groundwater can be motile^13^,^14^, however it has been observed that only a small fraction (<10%) of prokaryotes are motile in other well studied aquatic systems at any one time, likely due to energy limitations^15^,^16^. Aquifer systems are generally considered to be extreme environments due to the low levels of inorganic nutrient input, a lack of easily accessible organic carbon, lack of sunlight and low oxygen levels^17^, making energy limitations particularly relevant in these systems. Therefore, the dominant and fastest form of transport in groundwater ecosystems is likely due to passive advection, whereby prokaryotes are transported with the bulk motion of flowing groundwater, rather than by active motility. Within sand and gravel groundwater systems, flow rates typically range between 1 and 1000 meters per year^18^, making the rate of movement via advection for prokaryotes in groundwater slow. Here, we suggest that prokaryotic dispersal rates in groundwater may be significantly enhanced by direct attachment to invertebrates permanently living in the aquifer matrix, known as stygofauna.

The propensity of stygofauna to act as prokaryotic vectors in groundwater is currently unknown. It is been established in marine zooplankton that dispersal of prokaryotes in aquatic ecosystems is enhanced by direct association with eukaryotes^19^. The “hitch-hiking” prokaryotes associated with zooplankton undergo increased movement and the exploitation of more favourable conditions^19^. The ability for stygofauna to act as vectors for the enhanced movement of prokaryotes in groundwater ecosystems has not been investigated. Here, we assess the microbial communities associated with stygofauna in an aquifer ecosystem and determine how this association may enhance microbial transport in aquifer ecosystems.

## Results and Discussion

Due to the endemic oligotrophic conditions, microbes in groundwater systems are commonly found attached to surfaces where nutrient loads are higher^3^,^20^. Here, we suggest that stygofauna are an uncharacterised source of organic matter whose feeding, movement and excretion in groundwater systems are thought to mediate the transfer of organic matter through the aquifer system^21^. Consequently, “hitch-hiking” microbes have the potential to be transported throughout an aquifer system, while also remaining within close proximity to a source of organic matter.

Eleven Amphipoda, one Syncarida and three Oligochaeta specimens collected from a groundwater observation well in Mitchell Park, South Australia, were used in the experiments. Amphipoda were the most active and were therefore used for laboratory experiments to measure swimming speeds. The average swimming speed measured was 6.9 ± 4.6 × 10^4^ m yr^−1^ (Table 1). The average swimming speed of stygofauna was corrected for tortuosity, which permitted direct comparison with advective transport. Gravel and sand tortuosity corrections of 2 and 4^22^,^23^ resulted in stygofauna migration speeds between 1.7 and 3.5 × 10^4^ m yr^−1^ (Table 1). Individual stygofauna species are thought to have discrete habitats, restricted to single aquifers, with significant genetic structuring over short geographical distances^24^. However, other studies have shown that haplotypes can be shared across km ranges^25^,^26^,^27^, suggesting their movement is dependent on the properties of the regional aquifer matrix and will therefore vary. Within sandy and gravel groundwater systems, flow rates vary from site to site, however an average range of between 1 and 1000 meters per year^18^ is often observed, suggesting that if prokaryotes are moving via advection alone, they have the potential to move on average up to 1 km yr^−1^. Therefore, if the stygofauna are indeed travelling km ranges, our results suggest that prokaryotes associated with stygofauna have the potential to be transported 17–34× faster than those travelling with the bulk groundwater movement. These calculations however do not take into consideration that stygofauna are likely moving back and forth throughout the aquifer rather than swimming in one direction at all times. Stygofauna tracking experiments are therefore needed to fully understand stygofauna and transported microbe movement in groundwater ecosystems.

**Figure 1:**
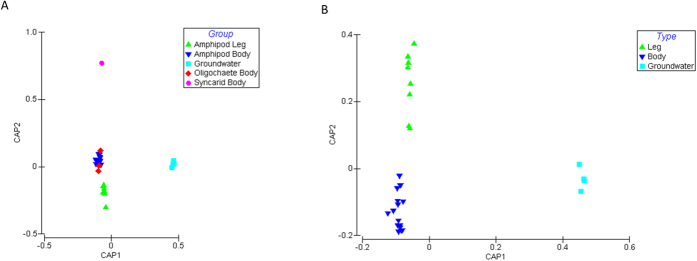
Comparison of the prokaryotes associated with stygofauna body, leg and groundwater samples. CAP analysis (using m = 11 principle coordinate axes) is derived from the sum of squared canonical correlations of 16S rRNA sequences matching the Greengenes database, order level. (**A**) Comparison of stygofauna groups bodies and legs, and the surrounding groundwater (**B**) Comparison of stygofauna bodies and legs and the surrounding groundwater.

Canonical analysis of principle coordinates (CAP)^28^ revealed a significant separation between the prokaryotic taxonomic composition on stygofauna bodies and legs, and the surrounding groundwater (*P-*value <0.0006; Fig. 1). A total of 96–97% of the data was explained by the hypothesis that there was a difference between stygofauna groups, bodies, legs and groundwater (Tables 2 and 3). Therefore, our data suggest that there are unique microbial communities between stygofauna groups, between the bodies and the legs of the Amphipoda and between the surrounding groundwater. For example, of all the prokaryotes isolated in this study, 31 orders were unique to the stygofauna and not isolated in the surrounding groundwater (Table S1). The majority of these have been previously associated with gut environments and so are likely derived from the stygofauna guts. *Nitriliruptorales*, *Thiohalorhabdales* and *Puniceicoccales*, however, have all been isolated from the environment indicating these may not be endemic to the stygofauna^29^,^30^,^31^. This suggests stygofauna also have the potential to transport gut and externally attached prokaryotes through the aquifer, which has major ecological impacts of community composition and dynamics. Furthermore, SIMPER analysis revealed an overrepresentation of unassigned prokaryotes on the stygofauna when compared to the surrounding groundwater (Table S2). This suggests that the prokaryotes associated with stygofauna may include a novel and uncharacterised niche for prokaryotes.

**Figure 2:**
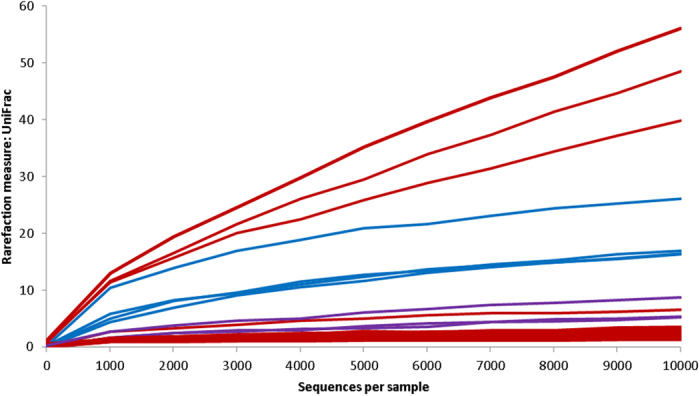
Rarefaction curves rarefied to 10,000 sequences for bacterial communities associated with the stygofauna and the groundwater. Each curve represents the overall bacterial 16S rRNA metagenome recovered from each stygofauna body, leg and the surrounding groundwater. The rarefaction curve, plotting the UniFrac rarefaction measure as a function of the sequences per samples, was computed in QIIME. Blue represents groundwater, red represents stygofauna bodies and purple represents Amphipoda legs.

There were also clear differences within the overall taxonomic diversity between the stygofauna bodies, legs and the surrounding groundwater, as indicated by the UniFrac rarefaction curves rarefied to 10,000 sequences (Fig. 2). The groundwater and Amphipoda leg diversity were all consistent in terms of their overall diversity. However, the stygofauna bodies varied broadly with only 3 of the 15 bodies exhibiting a higher diversity than the surrounding groundwater (Fig. 2). Differences in diversity attached to stygofauna bodies may be influenced by origin of the individual stygofauna, average rate of movement and overall prokaryotic abundance in the groundwater and/or on the stygofauna. This suggests that some “hitch-hiking” prokaryotes may be opportunistic rather than niche specific.

**Figure 3:**
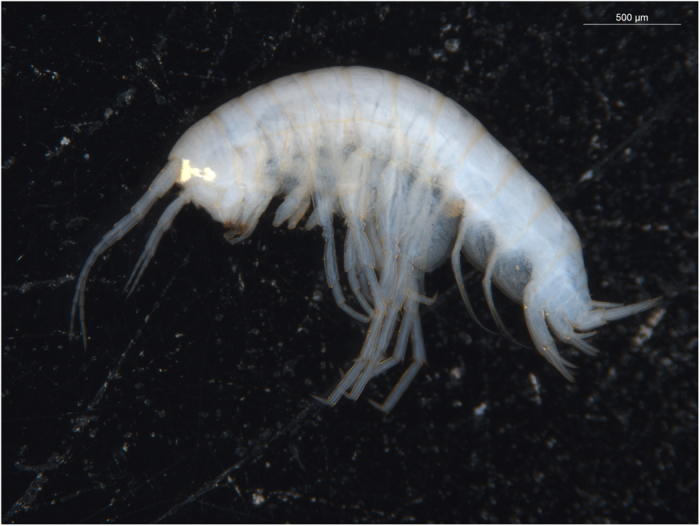
Undescribed species of stygobitic Neoniphargidae, Amphipoda, collected from the aquifer in Mitchell Park, South Australia.

The average length, width and height of the Amphipoda collected during the current study was 2.7 mm ± 0.3 mm, 0.5 mm ± 0.0 mm and 0.5 mm ± 0.0 mm, respectively (Table 1 and Fig. 3). Using NIS Elements software (AR4.5.00 64 bit), the total surface area was calculated to be 20 mm^2^ with a correction for the width of the legs. Based on an average prokaryote length of 1.25 μm and width of 0.36 μm from a mixed community^32^ it is possible that at least 4.4 × 10^7^ prokaryote cells are able to attach to an individual stygofauna body. A recent study investigating ultra-small prokaryotes in groundwater observed that bacteria can be up 56 times smaller in volume than the average estimate^33^, suggesting that this could increase bacterial attachment to 10^8^ prokaryotic cells. Flow cytometry counts revealed prokaryotic abundance of 2.4 ± 0.3 × 10^8^ cells L^−1^ in the surrounding groundwater, suggesting that the number of attached prokaryotes may be up to half an order of magnitude lower than the number of prokaryotes collected from the surrounding groundwater. These calculations however are likely to be an underestimate in that the attached prokaryotes are likely to form biofilms^3^,^13^. Therefore, it is likely that the number of prokaryotes attached to stygofauna outnumber those in the surrounding groundwater, warranting further investigation into the number of attached prokaryotes.

The relative volume of a stygofauna body based on the recorded average measurements equates to 0.675 μl. Based on flow cytometry counts, this stygofauna volume would represent approximately 10^2^ prokaryotes of the surrounding groundwater compared to the 10^7^ prokaryotes capable of attaching to a stygofauna body. This equates to an approximate 5 orders of magnitude difference in abundance between the stygofauna and the surrounding groundwater. Thus, our results indicate that stygofauna have the potential to transport up to 5 orders of magnitude more prokaryotes throughout the aquifer, 17–34× faster and greater distances than those travelling via advection alone in the bulk groundwater. Previous findings of “hitch-hiking” prokaryotes have shown that their association with migrating animals are an important mechanism for rapidly relocating microbes^19^. This suggests that stygofauna potentially have critical roles in transporting water purifying, remediating and pathogenic prokaryotes.

Here we demonstrate for the first time that “hitch-hiking” prokaryotes associated with stygofauna have the potential to be transported up to 34× faster, across km ranges and carrying 5 orders of magnitude greater abundance of prokaryotes when compared to transport via advection within bulk groundwater flow. Our microbial diversity measure also revealed prokaryotes associated with stygofauna can be at a higher diversity than those in the surrounding groundwater, likely due to bacterial attachment as an opportunistic survival mechanism in an oligotrophic environment. Therefore, stygofauna have the potential to influence overall community structure by transporting and introducing prokaryotes into other parts within the same aquifer. As prokaryotes underpin groundwater ecosystem dynamics^2^,^3^,^13^, influencing the prokaryotic make-up of a given environment has major ecological implications of potentially altering function. The preservation of groundwater is becoming increasingly important and so understanding the microbial dynamics that drive the system are crucial. Stygofauna represent a previously unexplained hotspot and transport mechanism for prokaryotes in groundwater and should be taken into consideration when assessing community dynamics in groundwater ecosystems.

## Methods

### Site Selection

Groundwater samples were sourced from a quaternary aquifer within the Pooraka formation, through a groundwater observation well located at Mitchell Park, Adelaide, South Australia (35°00′42.5″S 138°33′38.3″E) in May 2014 and March 2015. Site access granted by the Department of Environment, Water and Natural Resources (DEWNR). The Mitchell Park aquifer is a sandy/gravel aquifer system and was sampled at the depth of 12 m.

### Groundwater sampling

Groundwater was collected from a piezometer which consisted of a 80 mm diameter PVC casing and a slot depth of 8–14 m. Prior to collection, the bore was first purged of 3 bore volumes to ensure that the most representative and uniform water samples from the aquifer were collected. Groundwater was obtained using a submersible 12 V, 39 m Monsoon pump (Thermo Fisher Scientific). A total of 5 L of water from each sampling trip was collected for molecular analysis. For enumeration of prokaryotes, triplicate 1 mL groundwater samples were fixed with glutaraldehyde (0.5% final concentration) and incubated at 4 °C for 15 min, then quick frozen in liquid nitrogen and stored at −80 °C prior to flow cytometry analysis^34^,^35^.

### Stygofauna collection

Stygofauna were collected using sterile 7 cm diameter weighted plankton nets that repeatedly filtered the water column to ensure that material and fauna became dislodged from the walls and bottom of the well. Collected fauna, specifically from the orders Amphipoda and Syncarida and class Oligochaeta, were transported back to the lab alive and immediately sorted into 16.34 cm diameter petri dishes containing the groundwater they were collected from. Following video recording, the specimens were preserved in molecular grade absolute ethanol and stored at −20 °C prior to molecular work.

### Microbial enumeration of groundwater

Prokaryotes from the collected groundwater were identified and enumerated by flow cytometry using a FACSCanto II flow cytometer (Becton Dickinson). Triplicate samples were thawed and diluted 1:10 with filter TE buffer (10 mM Tris, 1 mM EDTA), stained with SYBR-I Green solution (Molecular Probes) and incubated in the dark for 10 minutes^34^,^36^. Fluorescent beads with a diameter of 1 μm (Molecular Probes) were added to each sample for an internal size and concentration standard^37^. Data for each sample was collected and analysed using FlowJo software (© Treestar) and differences in cell side scatter and SYBR-I Green fluorescence were used to discriminate prokaryotes (Gasol *et al.*^37^).

### Sample filtration, microbial community DNA extraction and sequencing

Following groundwater collection in May 2014, 3 × 1 L of groundwater was sampled for 16S rRNA sequencing. Groundwater was filtered through 5 μm membranes to remove sediment particles then microbial biomass was collected on a 0.22 μm membrane filter. Microbial community DNA from the groundwater was extracted using the PowerWater DNA Isolation Kit (MoBio laboratories, Inc., Carlsbad, CA, USA). Based on low variability between the initial triplicate data analysis from May 2014, 1 × 1 L of groundwater subsequently collected for analysis in March 2015.

Amphipod legs were dissected from their bodies to compare surface prokaryotes versus those associated with the external body and gut contents. The microbial community DNA associated with the stygofauna bodies (Amphipoda, Syncarida and Oligochaeta) were then isolated using the PowerSoil DNA Isolation Kit (MoBio laboratories, Inc., Carlsbad, CA, USA). The stygofauna legs were subjected to direct PCR where the legs from each stygofauna specimen were placed into a PCR tube containing 12.5 μl of KAPA HiFi HotStart ReadyMix, 1 μl each of forward and reverse 16S rRNA primers and 10.5 μl of sterile TA Buffer. The direct PCR cycle conditions used were 95 °C for 3 minutes; 25 cycles of 95 °C for 30 seconds, 55 °C for 30 seconds, 72 °C for 30 seconds; and a final extension time of 72 °C for 5 minutes. Prior to all stygofauna molecular work, all leg and body samples were air dried to ensure no ethanol remained. Genomic and amplicon DNA was assessed for concentration and quality using a Qubit Fluorometer (Qubit dsDNA HS Assay Kit; Life Technologies) and by 1.5% TBE agarose gel electrophoresis. High molecular weight DNA was then sent to the Flinders Genomics Facility (Adelaide, Australia) for 16S rRNA sequencing. Prokaryotic diversity amplicons were generated by amplification of the 16S rRNA gene using the primers 27F (5′-*TCGTCGGCAGCGTCAGATGTGTATAAGAGACAG*AGRGTTTGATCMTGGCTCAG-3′) and 519R (5′-*GTCTCGTGGGCTCGGAGATGTGTATAAGAGACAG*GTNTTACNGCGGCKGCTG-3′) with Illumina ligated overhang sequences in italics. The 2 step Illumina PCR amplification method was used^38^ with the Nextera XT Index Kit (Illumina Inc., San Diego. CA, USA). Sequencing was conducted on the Illumina MiSeq platform using the MiSeq Reagent Kit v3 (600 cycle; Illumina). All sequencing data used in this study are available at http://dx.doi.org/10.7910/DVN/IQJM9Z.

### Data analysis

A total of 11 video recordings of Amphipod swimming behaviour were collected for a period of 0.27–2.11 minutes using an 8 megapixel, 1080p HD, 30 frames per second camera. Individual Amphipoda movements were tracked over time and distance travelled (cm) recorded and transformed to speed (m y^−1^). Amphipoda velocities were corrected for the tortuosity, which is the ratio of the average distance that a molecule must travel to cover a direct distance^39^, using a ratio range of 2 to 4^22^,^40^ to directly compare to average groundwater diffusion speeds.

The Paired-End read merger (PEAR) v.0.9.5^41^ was used to pair the forward and reverse Illumina reads from each groundwater and stygofauna sample. Merged reads were processed using Quantitative Insights Into Microbial Ecology (QIIME) v.1.8.0^42^ and UPARSE^43^ as previously described^44^, however without the removal of singletons. The quality filtering criteria used was a minimum 200 bp in length, no mismatches in the primer sequences, no more than 6 ambiguous bases and a minimum quality score of 30. USEARCH^45^ was used to perform filtering of duplicate sequences and chimera removal. The remaining sequences were clustered into operational taxonomic units (OTUs) based on sequence similarity using *uclust* and Greengenes database (13_08) as a reference in QIIME with a minimum identity cut-off of 97%.

Differences in overall taxonomic composition between the groundwater and stygofauna body and leg samples were analysed using the PERMANOVA+ version 1.0.3 3 add-on to PRIMER^28^,^46^. Bray-Curtis similarity distance matrices were calculated for square-root transformed data. To determine whether there were any significant differences between microbial taxonomic compositions, canonical analysis of principle coordinates (CAP) on the sum of squared canonical correlations was used^28^. The *a priori* hypothesis that the prokaryotic composition between the stygofauna types, bodies and legs and the surrounding groundwater were different was tested in CAP by obtaining a *P*-value using 9999 permutations.

To compare the relative levels of prokaryotic OTU diversity within the stygofauna types, bodies versus legs and surrounding groundwater, UniFrac^47^ alpha diversity measure was used to generate rarefaction curves on rarefied abundance measures. To determine those taxa that were consistently driving the dissimilarity between the stygofauna (bodies and legs combined) and the surrounding groundwater, similarity percentage (SIMPER) analysis was used. A Diss/SD ration of greater than 1.4 was used to indicate key discriminating taxa^48^.

## Additional Information

**How to cite this article**: Smith, R. J. *et al.* Stygofauna enhance prokaryotic transport in groundwater ecosystems. *Sci. Rep.* **6**, 32738; doi: 10.1038/srep32738 (2016).

## Supplementary Material

Supplementary Table S1

Supplementary Table S2

## Figures and Tables

**Table 1:** Length and swimming speeds of stygofauna collected from the Mitchell Park aquifer.

Stygofanua Specimen	Length (mm)	Swimming Speed (×10^6^ m yr^−1^)						
		*Replicate 1*	*Replicate 2*	*Replicate 3*	*Replicate 4*	*Replicate 5*	*Average*	*St Deviation*
Amphipoda 1	2.5	12.68	11.75	7.77	10.91	4.30	9.48	3.44
Amphipoda 2	2.5	11.68	11.40	11.51	8.28	3.92	9.36	3.35
Amphipoda 3	2.5	5.25	3.91	4.19	4.37	4.10	4.36	0.52
Amphipoda 4	2.5	2.34	2.06	1.94	1.84	1.80	1.99	0.22
Amphipoda 5	2.5	10.32	11.51	8.74	14.84	13.03	11.69	2.36
Amphipoda 6	2.5	2.28	1.70	2.38	2.23	2.34	2.19	0.28
Amphipoda 7	2.5	11.64	21.89	13.36	14.27	12.08	14.65	4.18
Amphipoda 8	3.0	10.32	11.51	8.74	14.84	13.03	11.69	2.36
Amphipoda 9	3.0	2.70	6.89	1.99	2.47	2.45	3.30	2.02
Amphipoda 10	2.5	3.64	2.53	3.38	2.26	2.37	2.84	0.63
Amphipoda 11	3.5	6.18	1.95	5.35	2.72	3.42	3.93	1.78
Average	2.7						6.86	4.58
Standard Deviation	0.3							
Tortuosity minimum							3.43	
Tortuosity maximum							1.72	

**Table 2:** Results of CAP analysis testing the hypothesis that taxonomic composition of microbes at order level differ from samples collected from stygofauna type, stygofauna body, stygofauna legs and groundwater samples.

	Factor	m	Allocation Success % (ratio correct:misclassified)						δ^2^	*P*-value (δ^2^)	*P-*value (trace)
			Amphipod Body	Oligochaete Body	Groundwater	Amphipod Leg	Syncarid Body	Total			
**Taxonomy**	Order	11	100 (11:11)	100 (3:3)	100 (4:4)	87.5 (7:8)	100 (1:1)	96.3	0.99	**0.0007**	**0.0001**

**Table 3:** Results of CAP analysis testing the hypothesis that taxonomic composition of microbes at order level differ from between samples collected from stygofauna body, leg and groundwater samples.

	Factor	m	Allocation Success % (ratio correct:misclassified)				δ^2^	*P*-value (δ^2^)	*P-*value (trace)
			Stygofauna Body	Stygofauna Legs	Groundwater	Total			
**Taxonomy**	Order	11	100 (15:15)	100 (7:8)	100 (4:4)	96.3	0.99	**0.0001**	**0.0001**
